# Spatial Neurolipidomics at the Single Amyloid-β Plaque Level in Postmortem Human Alzheimer’s Disease Brain

**DOI:** 10.1021/acschemneuro.4c00006

**Published:** 2024-02-01

**Authors:** Wojciech Michno, Andrew Bowman, Durga Jha, Karolina Minta, Junyue Ge, Srinivas Koutarapu, Henrik Zetterberg, Kaj Blennow, Tammaryn Lashley, Ron M. A. Heeren, Jörg Hanrieder

**Affiliations:** † Department of Psychiatry and Neurochemistry, Sahlgrenska Academy, University of Gothenburg, Mölndal 43180, Sweden; ‡ Department of Neuroscience, Physiology and Pharmacology, University College London, London WC1E6BT, United Kingdom; § Department of Public Health and Caring Sciences, Uppsala University, Uppsala 75237, Sweden; ∥ Science for Life Laboratory (SciLife), Uppsala University, Uppsala 75237, Sweden; ⊥ Maastricht MultiModal Molecular Imaging Institute (M4I), Maastricht University, Maastricht 6229 ER, The Netherlands; # Clinical Neurochemistry Laboratory, Sahlgrenska University Hospital, Mölndal 43180, Sweden; ∇ Department of Neurodegenerative Disease, Queen Square Institute of Neurology, University College London, Queen Square, London WC1N 3BG, United Kingdom; ○ UK Dementia Research Institute at UCL, London WC1E 6BT, United Kingdom; ◆ Hong Kong Center for Neurodegenerative Diseases, Clear Water Bay, Hong Kong 999077, China; ¶ Wisconsin Alzheimer’s Disease Research Center, University of Wisconsin School of Medicine and Public Health, University of Wisconsin-Madison, Madison, Wisconsin 53726, United States; †† Paris Brain Institute, ICM, Pitié-Salpêtrière Hospital, Sorbonne University, Paris 75005, France; ‡‡ Neurodegenerative Disorder Research Center, Division of Life Sciences and Medicine, Department of Neurology, Institute on Aging and Brain Disorders, University of Science and Technology of China and First Affiliated Hospital of USTC, Hefei 230001, P. R. China; §§ Queen Square Brain Bank for Neurological Disorders, Department of Clinical and Movement Neurosciences, Institute of Neurology, University College London, London WC1N 1PJ, United Kingdom; ∥∥ Science for Life Laboratory (SciLife), University of Gothenburg, Gothenburg 40530, Sweden

**Keywords:** Alzheimer’s disease, β-amyloid, plaque pathology, neurolipidomics, mass spectrometry imaging, presenilin 1

## Abstract

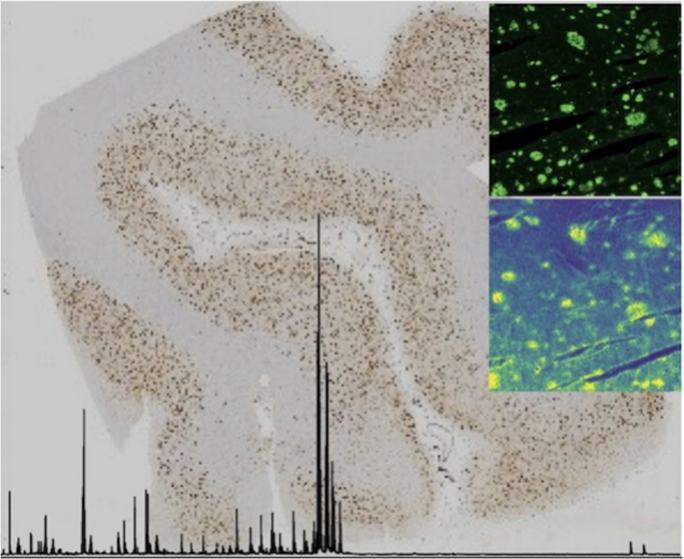

Lipid dysregulations have been critically implicated in Alzheimer’s disease (AD) pathology. Chemical analysis of amyloid-β (Aβ) plaque pathology in transgenic AD mouse models has demonstrated alterations in the microenvironment in the direct proximity of Aβ plaque pathology. In mouse studies, differences in lipid patterns linked to structural polymorphism among Aβ pathology, such as diffuse, immature, and mature fibrillary aggregates, have also been reported. To date, no comprehensive analysis of neuronal lipid microenvironment changes in human AD tissue has been performed. Here, for the first time, we leverage matrix-assisted laser desorption/ionization mass spectrometry imaging (MALDI-MSI) through a high-speed and spatial resolution commercial time-of-light instrument, as well as a high-mass-resolution in-house-developed orbitrap system to characterize the lipid microenvironment in postmortem human brain tissue from AD patients carrying Presenilin 1 mutations (PSEN1) that lead to familial forms of AD (fAD). Interrogation of the spatially resolved MSI data on a single Aβ plaque allowed us to verify nearly 40 sphingolipid and phospholipid species from diverse subclasses being enriched and depleted, in relation to the Aβ deposits. This included monosialo-gangliosides (GM), ceramide monohexosides (HexCer), ceramide-1-phosphates (CerP), ceramide phosphoethanolamine conjugates (PE-Cer), sulfatides (ST), as well as phosphatidylinositols (PI), phosphatidylethanolamines (PE), and phosphatidic acid (PA) species (including Lyso-forms). Indeed, many of the sphingolipid species overlap with the species previously seen in transgenic AD mouse models. Interestingly, in comparison to the animal studies, we observed an increased level of localization of PE and PI species containing arachidonic acid (AA). These findings are highly relevant, demonstrating for the first time Aβ plaque pathology-related alteration in the lipid microenvironment in humans. They provide a basis for the development of potential lipid biomarkers for AD characterization and insight into human-specific molecular pathway alterations.

## Introduction

Alzheimer’s disease (AD) is responsible for ∼70% of dementia cases. As the disease is believed to start more than 20 years before any clinical symptoms appear, there is a need for detailed phenomic characterization.^1,2^ The classical hallmarks of AD are amyloid-β (Aβ) plaque deposition, and formation of neurofibrillary tangles made up of hyperphosphorylated tau proteins. Consequently, disease-modulating strategies have historically focused on targeting the peptides involved in these pathologies, Aβ and tau. Lipids, however, have also been implicated in AD and more specifically in Aβ plaque formation.^3^ This is largely motivated by that the ε4 allele of the apolipoprotein E encoding gene (APOE), a lipid transporter, is the most prominent genetic risk factor for developing sporadic AD.^4^ Additionally, recent genome-wide association studies (GWAS) have identified multiple lipid-sensing or lipid transporter proteins associated genes as AD risk factors (e.g., TREM2, CLU, ABCA7).^5^

The relevance of lipid microenvironment alteration in the context of single Aβ plaques has also been demonstrated in transgenic AD mouse models. Here, Aβ plaque-specific changes in both sphingolipids and phospholipids have been reported using matrix-assisted laser desorption/ionization (MALDI)-based mass spectrometry imaging (MSI).^6−8^ Moreover, such changes were found to be tied to the structural polymorphism of individual Aβ plaques.^6,8^ Still, although unlikely, such lipid alterations could be caused by aberrant APP/Aβ peptide expression in the majority of the transgenic AD rodent models. Therefore, it is of essential interest to identify human-specific alterations in lipid metabolism that are associated with Aβ plaque pathology.

To advance our molecular understanding of lipid metabolic alterations in the context of Aβ pathology in human AD, we developed an analytical approach to study the chemical lipid composition of individual Aβ plaques in human AD. We specifically focused our analysis on the genetic form of AD, specifically subjects carrying *Presenilin* 1 mutations (PSEN1) leading to familial AD (fAD). Herein, we established an MSI protocol capable of sensitive lipid imaging at 10 μm across large regions of human brain tissue. Through these combined MSI approaches, we demonstrate localization/depletion of nearly 40 sphingolipid and phospholipid species on the level of single Aβ plaques. These findings are highly relevant, demonstrating for the first time Aβ plaque pathology-related alteration in lipid microenvironment specifically in human subjects.

## Methods

### Patient Characteristics

Human postmortem brain tissue was obtained through the brain donation program at Queen Square Brain Bank for Neurological Disorders (QSBB), Department of Clinical and Movement Neurosciences, Institute of Neurology, University College London (UCL).

The standard diagnostic criteria were used for the neuropathological diagnosis of AD.^9^ The demographic and neuropathological classifications are shown in Table 1. Temporal cortex tissue was used from all cases, collected at the QSBB in a routine manner where one hemisphere is fresh-frozen and the other formalin-fixed. Pathological assessment along with H&E and immune staining was carried out on the formalin-fixed hemisphere (Supporting Figure 1). Fresh-frozen tissue for mass spectrometry was used for the equivalent region taken from the frozen hemisphere.

**Table 1:** Patient Chart Summarizing the Demographics and Diagnostic Scores of PSEN1 Familial AD Cased Used in This Study

patient	mutation	sex	age at onset	age at death	duration	clinical diagnosis	pathological diagnosis	Braak tau	Thal phase	CERAD	ABC
patient 1	PS1 A434T & T291A	M	42	47	5	FAD	FAD	5	5	3	A3B3C3
patient 2	PS1 R278I	F	46	65	19	FAD	FAD	6	5	3	A3B3C3
patient 3	PS1 L250S	M	47	58	11	FAD	FAD	6	5	3	A3B3C3
patient 4	PS1 E120 K exon 5	F	31	37	6	FAD	FAD	6	5	3	A3B3C3
patient 5	PS1 E184D	F	45	58	13	FAD	FAD	6	5	3	A3B3C3

The study was conducted in accordance with the provisions of the Declaration of Helsinki and approved by the National Hospital for Neurology and Neurosurgery Local Research Ethics Committee, UCL, U.K. Ethical approval from a Swedish panel was received for the same experiments: DNr 012-t5; 150 416 (Göteborg). For this study, no randomization, blinding, preregistration, or sample size calculations were performed. No inclusion or exclusion criteria were applied.

### Chemicals and Reagents

All chemicals for matrix and solvent preparation were pro-analysis grade and obtained from Sigma-Aldrich (St. Louis, MO). TissueTek optimal cutting temperature compound was purchased from Sakura Finetek (AJ Alphen aan den Rijn, The Netherlands). The ddH_2_O was obtained from a Milli-Q purification system (Millipore Corporation, Merck Millipore, Billerica, MA).

### Immunohistological Staining of Fixed Tissue

One brain hemisphere was fixed in formalin and embedded in paraffin according to QSBB standard procedures. Representative images for each patient were acquired from 8-μm-thick sections that were deparaffinized and rehydrated using xylene and graded ethanol, respectively. Endogenous peroxidase activity was blocked by the addition of 0.3% H_2_O_2_ in methanol for 10 min. Tissue sections were pretreated in 100% formic acid for 10 min, washed, and further treated in citrate buffer (pH 6.0) for 10 min in a pressure cooker. Nonspecific binding was blocked with 10% dried milk solution. Incubation with the primary antibody (anti-Aβ, epitope amino acids 8–17), DAKO, 6E10 antibody (1:500) was performed for 1 h at room temperature (RT, 25 °C), followed by incubation with biotinylated antirabbit IgG for 30 min at 25 °C and avidin–biotin complex for additional 30 min. Chromogenic development was performed with diaminobenzidine/H_2_O_2_.

### Amyloid Fluorescence Staining

Prior to MALDI analysis, fluorescent plaque imaging was performed using luminescent conjugated oligothiophene (LCO) amyloid probes.^10^ In brief, sections were fixed in absolute EtOH for 8 min, partially rehydrated in 70% EtOH for 30 s, and rinsed twice in phosphate buffer solution (PBS) for 30 s. For amyloid staining, 30 min incubation with heptamer-formyl thiophene acetic acid (h-FTAA) (1.5 μM) was used.^10,11^ Following staining, tissue was washed three times for 1 min in PBS and dried at 25 °C. Overview imaging was performed using a wide-field microscope (Axio Observer Z1; Zeiss) with a Plan-Apochromat 10 × /0.3 DIC objective and a 38 HE-AF488 filter (Ex: BP 479/40; Em: BP 525/50). Prior to MALDI-MSI analysis, fluorescent images were imported into FlexImaging (v 5.0; Bruker Daltonics, Bremen, Germany) software at 25% size compression in order to guide MALDI-MSI analysis and (post-MALDI-MSI analysis) into SCiLS Lab (v2019; Bruker Daltonics, Bremen, Germany).

### MALDI Sample Preparation and Matrix Application

For MALDI imaging of lipids, 10 μm thick tissue cryo-sections were washed in 10 mM ammonium formate (AmF) followed by application of 1,5 diamino-naphthalene (1,5-DAN) matrix using a TM-sprayer (HTX Technologies, Carrboro, NC). Before spraying, the solvent pump (Dionex P-580, Sunnyvale, CA) was purged with 70% ACN at 500 μL/min for 10 min followed by a manual rinse of the matrix loading loop using a syringe. A matrix solution of 20 mg/mL 1,5-DAN in 70% ACN was sprayed onto the tissue sections with the following instrumental parameters: nitrogen flow (12 psi), spray temperature (80 °C), nozzle height (40 mm), five passes with offsets and rotations, spray velocity (1250 mm/min), and isocratic flow of 50 μL/min using 70% ACN as the pushing solvent.

### Mass Spectrometry Imaging Analysis

High-speed MALDI-MSI acquisition was performed at 10 μm spatial resolution by using a MALDI-TOF instrument (rapifleX, Bruker Daltonics). The MALDI source is equipped with a scanning Smartbeam three-dimensional (3D) laser featuring a laser beam diameter of 5 μm. Spectra were acquired by using custom laser settings with a resulting field size of 10 μm. The measurements were performed with the laser operating at a frequency of 10 kHz with 20 laser pulses per pixel. Acquisition and subsequent processing were performed by using the instrument software FlexImaging 5.0 (Bruker Daltonics). Acquisition of high-mass-resolution MSI data was performed by using an Orbitrap Elite mass spectrometer (Thermo Fisher Scientific GmbH, Bremen, Germany) coupled to a reduced-pressure ESI/MALDI ion source (Spectroglyph LLC, Kennewick, WA). Further details on the ion source can be found in the literature.^12^ The 349 nm MALDI laser (Spectra Physics, Mountain View, CA) was operated at a repetition rate of 1000 Hz and pulse energy of ∼1.5 μJ. The laser was focused to a spot size/step size of ∼20 × 20 μm^2^, mass resolution was chosen to be 120,000 (at *m*/*z* 400), and the total scan time was 1.05 s/scan and pixel.

### Data Processing and Statistical Analysis

The MALDI-TOF MSI data analysis was performed in SCiLS Lab (v. 2019, Bruker Daltonic). The MALDI imaging data were total ion current (TIC) normalized. Cluster analysis-based spatial segmentation (bisecting *k*-means) was used to identify characteristic lipid distributions and for region of interest (ROI) annotation. Nearly 100 Aβ plaques were annotated as ROIs for each of the patients. The Aβ plaque ROIs were exported as *.csv. This was followed by a binning analysis for data reduction. Here, all ROI data were imported into Origin (v. 8.1 OriginLab), and peaks and peak widths were detected on the average spectra of each ROI using the implemented peak analyzer function. Bin borders for peak integration were exported as tab-delimited text files and were used for area under curve peak integration within each bin (peak-bin) of all individual ROI average spectra using an in-house-developed R script. Single pixel signal correlation (SPSC) of individual lipid signals for each of the identified species was performed directly in SCiLS Lab (v. 2019, Bruker Daltonic).

The orbitrap data visualization and data analysis were performed using LipostarMSI (Molecular Horizon Srl, Bettona, Italy). MSI *.raw data (Thermo Fisher Scientific GmbH, Bremen, Germany) were converted into imzML^13^ by first converting *.raw data into mzML using msconvert (ProteoWizard).^14^ Using the built-in converter of LipostarMSI, the mzmL file was then combined with the positioning file created by the MALDI/ESI injector to generate a profile-mode imzML file. Lipid identification within LipostarMSI was performed with reference to the LIPIDMAPS database^15^ and was based on accurate *m*/*z* matching using a tolerance of ±2 ppm and reference to the literature.^16^ Phospholipids, sphingolipids, and sterols were considered for identification.

## Results

### Mass Spectrometry Imaging Delineated Lipid Microenvironment of Individual Aβ Plaques in Human AD Brain Tissue

**Figure 1:**
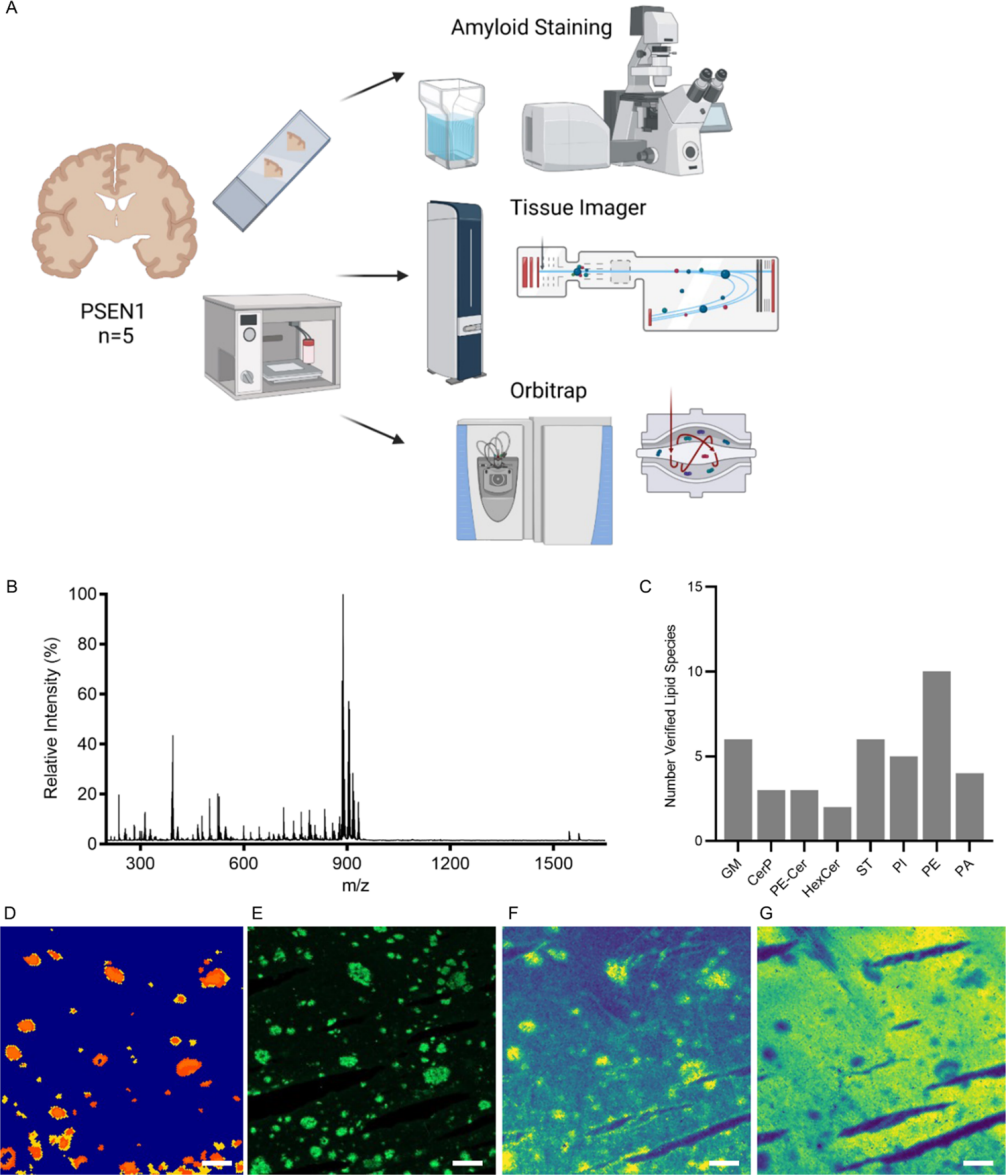
Setup for chemical analysis of sphingolipids and phospholipids in postmortem human AD tissue of patients with *PSEN1* mutations. (A) Schematic of the setup of this study where frozen postmortem tissue from five *PSEN1* mutation carrying patients was consecutively cryo-sectioned on conductive ITO glasses and used for either LCO amyloid staining to identify Aβ plaque pathology (top) or sprayed with a chemical matrix for lipid analysis using tissue-imager rapifleX (middle) or high-mass-resolution orbitrap (bottom) (Created with BioRender.com). (B) Average mass spectra of Aβ plaques from one of the *PSEN1* patients. (C) Bar plot representing the numbers of verified lipid species grouped by subtype. (D) Spatial segmentation by k-means clustering of rapifleX data allowed for the identification of Aβ plaque-like regions of interest (ROIs). The identity of these ROIs as Aβ plaques was confirmed through (E) LCO amyloid staining. This staining (E) overlapped well both with the clustering (D), but even better single ion images of enriched species such as (F) monosialo-ganglioside GM1(d18:1/20:0), and depleted species such as (G) sulfatide ST(d18:1/24:0). Scale bar: 150 μm.

To study the lipid microenvironment of Aβ plaque pathology, we established a mass spectrometry imaging approach allowing for 10 μm analysis of fresh-frozen postmortem human brain tissue. Following sequential cryo-sectioning of five fAD–PSEN1 subjects, we stained one of the adjacent sections using amyloid-specific LCO probes (Figure 1A (top)). This staining was used as guidance for the identification of Aβ plaque-rich regions for subsequent MSI experiments. Further, this allowed us to later verify that the observed MALDI-MSI signal could indeed be attributed to microenvironmental changes that are local to the Aβ plaque pathology. Then, the adjacent glass slide was coated with 1,5-DAN matrix followed by lipid imaging using MALDI-TOF MSI (rapifleX, Figure 1A (middle)).

To verify the putatively assigned lipid species, high-mass-resolution MALDI-MSI analysis was performed on an Orbitrap Elite mass spectrometer coupled to a reduced-pressure ESI/MALDI ion source (Figure 1A (bottom)).

Initial inspection of the mass spectra acquired from the AD patient’s postmortem brain tissue revealed a rich diversity of lipid species (Figure 1B). We performed spatial segmentation using cluster analysis (bisecting *k*-means) to delineate lipid signature of individual Aβ plaques that distinguish these pathological features from the local tissue lipid environment. Indeed, the clustering analysis identified unique Aβ plaques resembling features (Figure 1D). To verify the nature of these putative amyloid plaque features, we aligned the MALDI-MSI pseudocluster images of individual patients with the corresponding fluorescent microscopy images collected from adjacent tissues that were stained with amyloid-specific fluorescent probes (LCO, Figure 1E). Here, a high correlation between both MALDI-MSI and amyloid-specific fluorescent staining data was observed. We therefore proceeded with defining the identified pseudoclusters as regions of interest (ROIs) corresponding to Aβ plaques. These plaque ROI contained species that were both enriched (e.g., GM1(d18:1/18:0)) (Figure 1F) or depleted (e.g., ST(d18:1/24:0)) (Figure 1G). Interrogation of the *m*/*z* values underlying the pseudoclusters obtained from *k*-mean clustering demonstrated localization of nearly 100 species that were either enriched or depleted in the Aβ plaque ROIs. We were able to verify the identity of nearly 40 of these species using high-mass-resolution MALDI orbitrap (Table 2). Here, between 2 and 10 species of GM, HexCer, CerP, PE-Cer, ST, PI, PE, and PA species were identified (Figure 1C).

**Table 2:** List of Lipid Species Verified Using High-Mass-Resolution MALDI Orbitrap^a^

lipid class	common lipid name	theor. mass	observed mass [M – H]^−^ (MALDI-TOF)	observed mass [M – H]^−^ (Orbitrap)	detected in animal model CNS
CerP	CerP(d18:1/16:0)	617,4784	616,453	616,4712	yes^17^
CerP	CerP(d18:1/18:0)	645,5097	644,421	644,5025	yes^8,17^
CerP	CerP(d18:1/20:0)	673,5410	672,483	672,5339	yes^18^
Cer-PE	PE-Cer(36:1)	688,5519	687,496	687,5443	yes^8^
Cer-PE	PE-Cer(38:1)	716,5832	715,565	715,5757	yes^8,17^
Cer-PE	PE-Cer(40:1)	744,6145	743,509	743,6070	yes^19^
GM	GM3(d18:1/18:0)	1180,7445	1179,599	1179,7366	yes^7,16,17,20,21^
GM	GM3(d18:1/20:0)	1208,7758	120,763	1207,7683	yes^17,22^
GM	GM2(d18:1/18:0)	1383,8238	1382,717	1382,8153	yes^7,8,16^
GM	GM2(d18:1/20:0)	1411,8551	1410,756	1410,8457	yes^23^
GM	GM1(d18:1/18:0)	1545,8767	1544,733	1544,8679	yes^16,17^
GM	GM1(d18:1/20:0)	1573,9080	1572,818	1572,8992	yes^16,17^
HexCer	HexCer(d18:1/12:0)	643,5023	642,394		yes^24^
HexCer	HexCer(18:1/14:0)	671,5336	670,464		yes^8^
PA	LPA(18:0)	420,2641	419,251	419,2569	yes^8^
PA	PA(16:0/16:0)	648,4730	647,445		yes^16^
PA	PA(16:0/18:1)	672,4730	673,441	673,4813	yes^16^
PA	PA(18:0/22:6)	748,5043	747,469	747,4970	yes^25^
PE	PE(18:0)	481,3168	480,245	480,3096	yes^7^
PE	PE(22:6)	525,2855	524,247	524,2785	yes^25^
PE	PE (18:1/18:0)	745,5622	744,523	744,5548	yes^16^
PE	PE(18:1/20:0)	773,5935	772,467		yes^16^
PE	PE(P-18:0/20:4)	751,5516	750,525	750,5444	yes^17^
PE	PE(18:0/20:4)	767,5465	766,559	766,5395	yes^16^
PE	PE(16:0/22:6)	763,5152	762,403	762,5078	yes^16^
PE	PE(P-18:0/22:6)	775,5516	77,447	774,5444	yes^23^
PE	PE(18:0/22:6)	791,5465	790,449	790,5392	yes^16^
PI	PI(16:0)	572,2962	571,242		yes^17^
PI	PI(18:0)	600,3275	599,274	599,3203	yes^16,17^
PI	PI(20:4)	620,1377	619,258	619,2890	yes^26^
PI	PI(16:0/20:4)	858,5258	857,414	857,5185	yes
PI	PI (18:0/20:4)	886,5571	885,468		yes^7,17,21^
ST	ST(d18:1/22:0)	863,6156	862,513	862,6080	yes^23^
ST	ST(d18:1/22:0(2OH))	879,6106	878,539	878,6033	yes^21^
ST	ST(d18:1/24:1)	889,6313	88,854	888,6238	yes^7,16,23^
ST	ST(d18:1/24:0)	891,6469	890,559	890,6398	yes^7,23^
ST	ST(d18:1/24:1(2OH))	905,6262	904,543	904,6187	yes^7,21,23^
ST	ST(d18:1/24:0(2OH))	907,6419	90,657	906,6346	yes^7,22,23^

a These species have been previously reported in different animal models’ CNS using MALDI-based imaging mass spectrometry (MSI) or liquid chromatography–tandem mass spectrometry (LC-MS) (marked as *).

### Human Aβ Plaques Are Associated with Alterations in Sphingolipids

**Figure 2:**
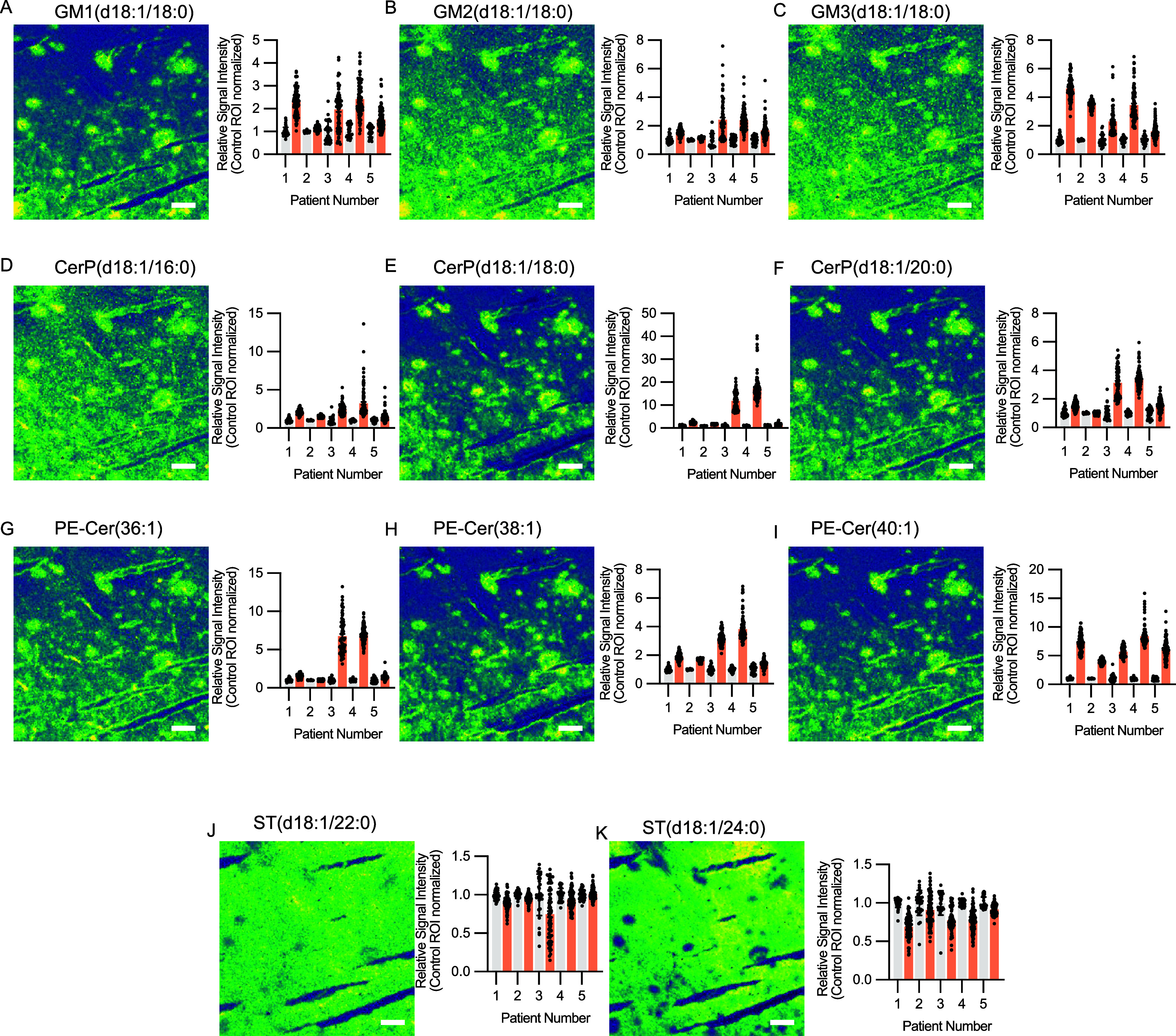
MALDI-MSI rapifleX data of sphingolipids in postmortem human AD tissue of five AD patients with *PSEN1* mutations. The Aβ plaque pathology is associated with local enrichment and depletion of several sphingolipids as apparent by the single ion images (left) and corresponding single Aβ plaque relative signal enrichment (right) of monosialo-gangliosides (GM), including (A) GM1(d18:1/18:0), (B) GM2(d18:1/18:0), and (C) GM3(d18:1/18:0). Similar Aβ plaque pathology-specific enrichment is also present for ceramide-1-phosphates (CerP) and ceramide phosphoethanolamine conjugates (PE-Cer), including (D) CerP (d18:1/16:0), (E) CerP (d18:1/18:0), and (F) CerP (d18:1/20:0) and (G) PE-Cer(36:1), (H) PE-Cer(38:1), and (I) PE-Cer(40:1), respectively. In contrast, an Aβ plaque specific depletion was observed for sulfatides, including (J) ST(d18:1/22:0) and (K) ST(d18:1/24:0). Signal intensities from 80 to 100 Aβ plaques and surrounding area was extracted for each patient. The red bar indicates signal from individual Aβ plaque ROIs, and gray corresponds to individual control ROIs. Scale bar: 150 μm.

Previous studies using transgenic AD mouse models have demonstrated Aβ plaque-specific enrichment and depletion of sphingolipid species, including GM, HexCer, CerP, PE-Cer, and ST. To date, alterations of these species have not been demonstrated in human AD tissue at the level of a single Aβ plaque. Herein, inspection of single ion images of verified lipid species along with the bar plots of lipid signal enrichment for individual plaques revealed a general plaque-associated enrichment of GMs, including GM1, GM2, and GM3 species with C18:0 (Figure 2A–C) and C20:0 (Supporting Figure 2A–C) fatty acid (FA) moieties. Enrichment of these was significantly higher in Aβ plaques as compared to control areas (Supporting Figures 3A–C and 4A–C). Additionally, we also observed the enrichment of a few neutral glycosphingolipids, including HexCer(30:1) and HexCer(32:1) (Supporting Figure 2D,E). Here, although a prominent plaque associated increase was apparent for all of the HexCer species within each patient, it was only significant for HexCer(32:1) on a group level (i.e., across patients) (Supporting Figure 4D,E). Lastly, phospho-ceramides, including CerP (d18:1/16:0), CerP (d18:1/18:0), and CerP (d18:1/20:0) (Figure 2D–F), as well as ceramide phospholipid conjugates, including PE-Cer(36:1), PE-Cer(38:1), and PE-Cer(40:1) (Figure 2G–I), were specifically enriched in Aβ plaque regions. As for HexCer, a clear plaque associated increase was present for all of the CerP and PE-Cer species within each patient. Though only a significant increase on a group level was determined for CerP(18:1/16:0), PE-Cer(38:1), and PE-Cer(40:1) (Supporting Figure 3D–I). In accordance with previous reports from mice,^6,8^ the enrichment of these sphingolipids species was accompanied by a general depletion of signal corresponding to multiple sulfatides (ST, Figure 2J,K) and their hydroxylated isoforms (ST–OH) (Supporting Figure 2F,G). Although a trend in depletion was present for all of the species, the only significantly reduced sulfatide was ST(d18:1/24:0) (Supporting Figure 3J,K and 4F,G).

### Human Aβ Plaque Are Associated with Alterations of Phospholipids

**Figure 3:**
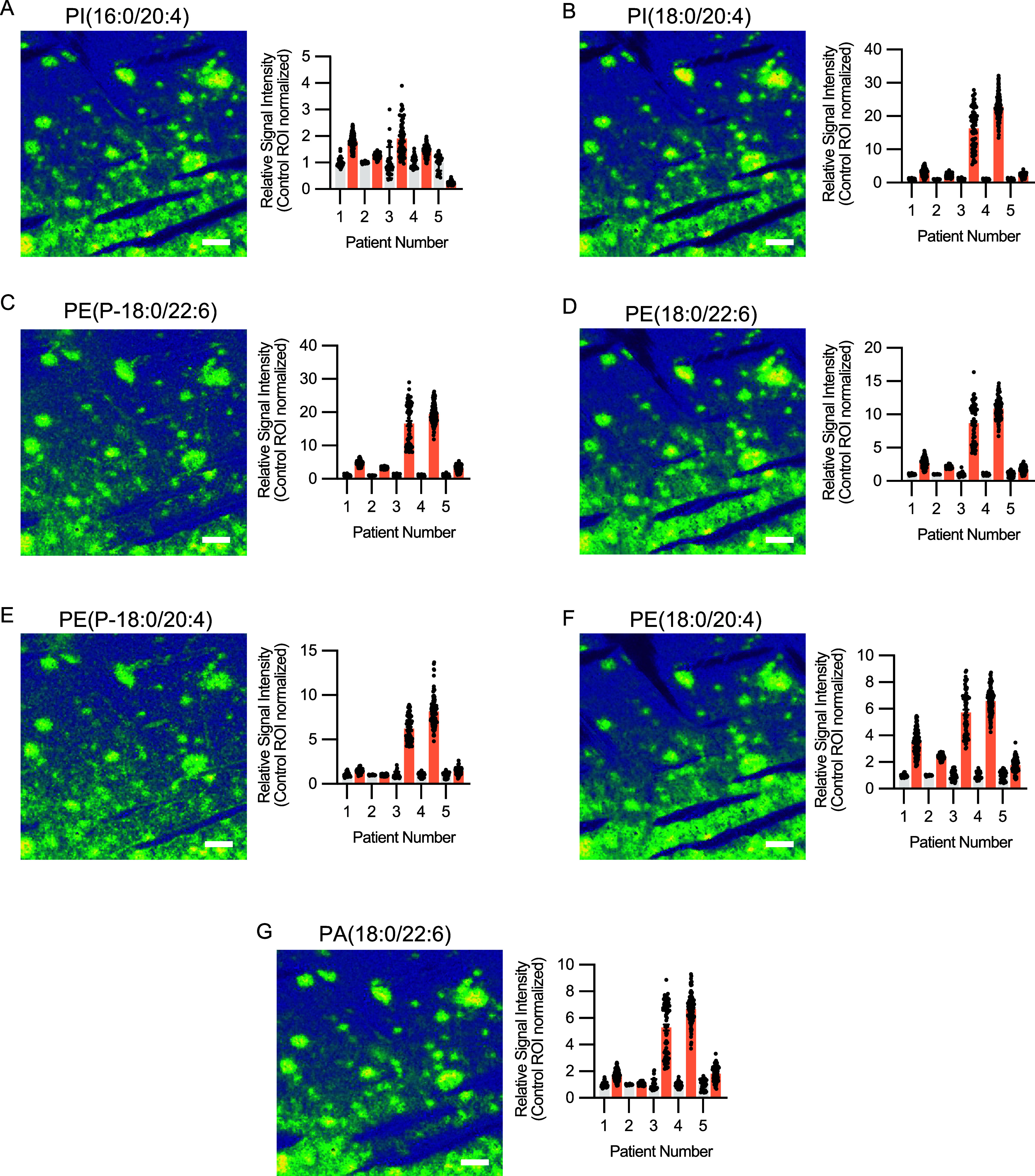
MALDI-MSI rapifleX data of phospholipids in postmortem human AD tissue of five patients with *PSEN1* mutations. The Aβ plaque pathology is associated with alterations of arachidonic acid (AA) or docosahexaenoic acid (DHA) containing phospholipids as apparent by the single ion images (left) and corresponding single Aβ plaque relative signal enrichment (right). This includes arachidonic acid (AA) residue containing phosphatidylinositoles, such as (A) PI(16:0/20:4) and (B) PI(18:0/20:4). Similar Aβ plaque pathology-specific enrichment is also present for DHA-containing phosphatidylethanolamine (C) PE(18:0/22:6) and plasmogen (D) PE(P-18:0/22:6), as well as the AA-containing (E) PE(18:0/20:4) and (F) PE(P-18:0/20:4). We also observed enrichment of DHA-containing phosphatidic acid, PA(18:0/22:6). Signal intensities from 80 to 100 Aβ plaques and surrounding area was extracted for each patient. The red bar indicates signal from individual Aβ plaques ROIs, and gray corresponds to individual control ROIs. Scale bar: 150 μm.

We next set to investigate whether the phospholipid microenvironment changes suggested from transgenic mice studies^6,7,16,20^ were also reflected in Aβ plaques in human brain. Indeed, inspection of segmentation loadings, the corresponding single ion images and bar plots revealed that the observed PI, PE, and PA were present as both lysophospholipids (Lyso-), and polyunsaturated fatty acid (PUFA) containing species, which was consistent with the animal studies (Table 2). However, although the majority of the PUFA-species had arachidonic acid (AA)- or docosahexaenoic acid (DHA) residues, we observed clear differences in what specific lipid subspecies with AA/DHA localized to the Aβ plaque pathology in the human subjects as compared to what has been reported for mice. In previous mouse studies, we observed enrichment of Lyso-PIs, specifically LPI(16:0) and LPI(18:0) (Supporting Figure 5A,B). However, these human samples instead show enrichment of LPI(20:4) (Supporting Figure 5C). Again, although the trend of Aβ plaque-specific enrichment was present for all of the species, it was significant only for the LPI(16:0) species (Supporting Figure 6A–E). Further, just as for transgenic mice, we also observed AA-containing PI(16:0/20:4) and PI(18:0/20:4), but in difference to mice, there was a lack of DHA-containing PI species (Figure 3A,B). Although enriched, none of the species was significantly increased in Aβ plaques (Supporting Figure 7A,B).

We next investigated the PE species present in human tissue, including PE plasmalogens (PE-P). Consistent with mice data we observed Lyso-PE species, specifically LPE(18:0) and LPE(22:6) (Supporting Figure 5D,E). Although enriched, none of the species were significantly increased in Aβ plaques on a group level (Supporting Figure 6D,E). Among the PE species, we observed plaque-specific enrichment as previously reported in transgenic mice, including PE(18:1/18:0), and PE(18:1/20:0) (Supporting Figure 2F,G). Here the PE(18:1/20) was significantly increased but not PE(18:1/18:0) (Supporting Figure 6F,G). We also observed DHA-containing PE(18:0/22:6) (Figure 3C) and the corresponding plasmalogen PE(P-18:0/22:6) (Figure 3D) to be enriched with Aβ plaques. Although the enrichment pattern was prominent (*p* = 0.07 and 0.08), it was not significant on the group level (Supporting Figure 7C,D). Additionally, we observed PE(16:0/22:6) (Supporting Figure 5H), which was nearly significant (*p* = 0.05) (Supporting Figure 6H). Consistent with the pattern of the PIs, we observed the AA-containing PE species not previously reported in mice. This included both PE(18:0/20:4) and PE(P-18:0/20:4) (Figures 3E,F and Supporting Figure 7E,F).

Lastly, in the context of human plaque pathology, we observed cyclic phosphatidic acid (CPA), for the first time, specifically CPA(18:0) (Supporting Figure 5I). Consistent with animal studies, we further observed PA(16:0/16:0) (Supporting Figure 5J) to localize to plaques. Although again increased, none of these was significant (Supporting Figure 6I,J). However, in contrast to the animal studies, we did not observe any AA-containing PA species but instead found increased but not significant plaque localization of DHA-containing PA(18:0/22:6) (Figure 3G) and (Supporting Figure 7G).

### Aβ Plaque-Associated Sphingolipids and Phospholipids Correlate within Lipid Class

**Figure 4:**
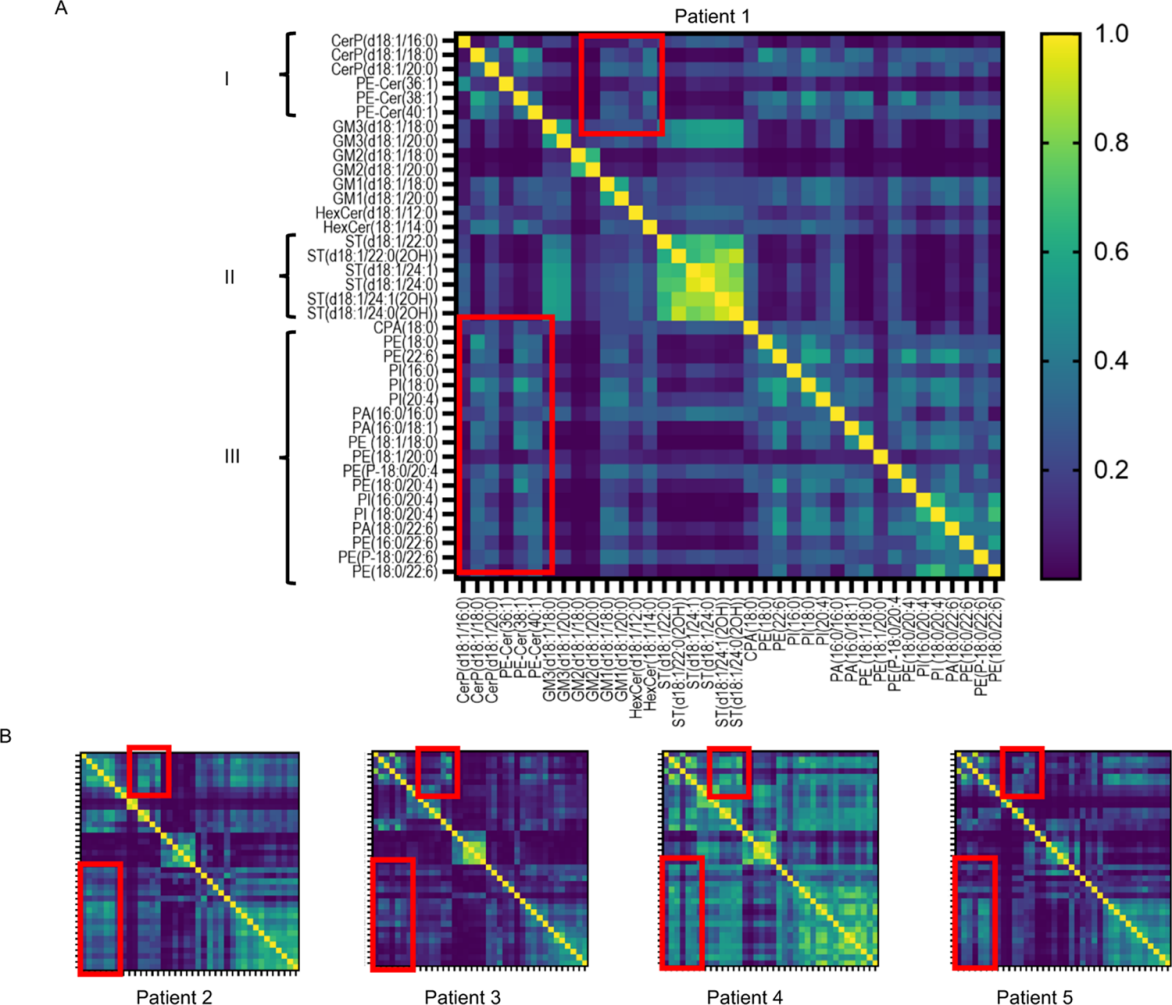
Heatmap representing single pixel signal correlation (SPSC) between individual sphingolipid and phospholipid for all of the *PSEN1* mutation carriers. (A, B) Similar correlation patterns were present among the patients, but with varying degree of correlation strength. (A-I) We observed an interesting correlation pattern of ceramide-1-phosphates (CerP) and ceramide phosphoethanolamine conjugates (PE-Cer) with (A-I, top red box) not only ceramide monohexosides (HexCer) but also the longest monosialo-gangliosides (GM), the GM1 species. (A-II) There was a colocalization pattern between sulfatides (ST), both in the hydroxylated and nonhydroxylated forms. (A-III) General correlation among phospholipid species that was strongest for those species that contain the arachidonic acid (AA) or docosahexaenoic acid (DHA) residues, (A-III, bottom red box). Further, there was some colocalization of the phospholipid species with sphingolipid species, in particular CerP and PE-Cer.

In mice, chemical diversity within individual plaques has been reported, down to the level of individual lipid species.^6,8,16,21,27^ Therefore, we set out to identify any lipid species that colocalize more/less with one another. This would presumably indicate enrichment/depletion of specific metabolic pathways during distinct stages of Aβ-plaque pathology progression. We performed SPSC within the areas corresponding to Aβ plaques for each of the fAD PSEN1 patients. The correlation analysis revealed a general diversity of lipid colocalization patterns between patients (Figure 4A,B). Still, a broad pattern among the CerP and PE-Cer species could be observed in all of the patients. This colocalization pattern did to some extent correlate with HexCer, and to our surprise also GM1 species (Figure 4A–I, top red box). As suggested by the single ion images and corresponding bar plots, a unique colocalization pattern was present among the ST species (Figure 4A–II). The correlation of ST(d18:1/22:0) and its hydroxylated form was not as strong as among the other ST species. Lastly, there appeared to be a general colocalization of the phospholipid species, which appeared strongest for the AA and DHA residues containing PI, PE, and PA species (Figure 4A–III). Interestingly, PUFA moieties showed some colocalization with sphingolipid species, in particular CerP and PE-Cer (Figure 4A–III, bottom red box).

## Discussion

It has been previously suggested that lipids play a central role in AD pathogenesis.^3^ AD-linked change in the levels of various lipid subclasses have been observed in cerebrospinal fluid, blood, and post-mortem human brain tissue extracts.^3,28,^ Still, none of these studies provide direct insight into possible local microenvironment changes that take place at the sites of Aβ plaque development. To overcome these limitations, chemical imaging approaches based on mass spectrometry have been developed. The majority of these approaches rely on MALDI-MSI which provides the molecular and spatial specificity necessary to delineate the complexity of lipid changes that take place in tissue at the cellular scale. Until recently, these approaches have only been successfully applied in transgenic AD mouse models,^7,16^ and therefore do not negate the need for the analysis of post-mortem human AD tissue.

In the current study, we performed pioneering analysis and verification of lipid microenvironment changes in post-mortem human AD tissue from patients carrying *PSEN*1 mutations. In detail, following comprehensive lipid analysis of post-mortem brain tissue from five *PSEN*1 mutation carrying AD patients,^30^ we verified the identity of these putatively assigned lipid species through complementary analysis using high-mass-resolution MALDI orbitrap instrumentation. Although we observed large differences in lipid signal enrichment within individual Aβ plaques between patients (see bar plots for respective single ion images), when considering individual patients, the lipid signal was normally distributed and consistent within Aβ plaque ROIs. This is emphasized by both the bar plots from individual Aβ plaques, average Aβ plaque, and control area per patient plots in Supporting Figures, and the single pixel signal correlation (SPSC) for individual patients (Figure 4). The patterns could possibly be clearer if the post-mortem tissues used in this study were from patients carrying the same *PSEN*1 mutations, which was not the case (Table 1) and which is a limitation of the current study. Indeed, differences in the age of onset of clinical symptoms and Aβ plaque pathology distribution in the brain have been reported for various *PSEN*1 mutations.^30,31^ However, a consistent pattern of lipid enrichment toward plaques was observed across all patients as represented by single ion images for major lipid species from each of the five patients (Supporting Figure 8).

In agreement with previous animal studies (Table 2), we observed GM, HexCer, CerP, PE-Cer, and ST, as well as PI, PE, and PA species, in association with plaque pathology. Of interest, plaque-associated ganglioside and sulfatide patterns have been reported for AD mouse models. Indeed, GM1 has previously been implicated in promoting and altering amyloid aggregation^32,33^ as well as modulating Aβ secretion through interaction with γ secretase complex.^34^ Plaque-specific depletion of sulfatides likely indicate demyelination.^23^ The phospholipid species that showed significant plaque localization comprised both intact and lysoforms (PA, PI, PE and LPI, LPA, LPE). Interestingly, anionic phospholipids have been implicated in plaque pathology through microglial signaling and activation through triggering receptor expressed on myeloid cells 2 (TREM2), as those lipids are TREM2 agonists.^35^ To further substantiate the single plaque localization patterns, we performed single pixel signal correlation across plaque ROI. Here, despite the wide diversity in for different lipid species, we observed a general strong correlation pattern across all patients for sphingolipids (CerP, PE-Cer, HexCer, and GM1) further substantiating their role in plaque pathology (Figure 4).

For (lyso) phospholipid species, a general correlation that appeared strongest among the AA- and DHA-containing species was observed (Figure 4).

The majority of the lipids found associated with plaque pathology overlapped between those from previous animal studies and the human tissue analyzed here (Table 2). However, a clear difference was observed in the number of AA- and DHA-containing species associated with plaque pathology in human brain tissue as compared to AD mouse models. Additionally, the presence of these two fatty acid configurations depended on lipid subtype. Specifically, we observed almost exclusively AA-containing PIs, and AA-based lyso-PI. For PAs, we observed mainly DHA-containing species. For PEs both AA and DHA species were observed. Both AA and DHA are precursors of eicosanoids, which are essential for mediating inflammatory mechanisms of both astro- and microglia.^36^ While AA is generally considered proinflammatory, DHA is believed to have the opposite effect. Interestingly, in the context of AD, recent evidence suggests that omega-3 fatty acids are involved in the modulation of synaptotoxicity mediated by microglial processes, where TREM2 appears to play an essential role^37,38^

Although our study does not delineate the exact role that distinct fatty acid residues and the lipid subtypes play in the molecular pathways of AD, the results indicate that lipid subtype-specific PUFAs are involved in Aβ plaque pathology. Likewise, it also confirms the previously proposed role of sphingolipids in AD plaque pathology.

It is important to emphasize that this study is largely descriptive in nature, and hence, no clear conclusion can be drawn, with respect to the limited number of cases used. Additionally, no elucidation of the lipid isomers was performed. Still, the results obtained here will guide subsequent examinations and act as a demonstration of novel analytical approaches that are highly relevant for lipid studies of proteopathies.

## Conclusions

In summary, this work is the first study of lipid microenvironment changes related to Aβ plaque pathology in post-mortem human AD tissue. Here we provide spatially resolved single ion patterns of multiple lipid classes (GM, CerP, PE-Cer, ST, PI, PE, and PA) localizing to Aβ plaques. In addition to previous transgenic AD mouse studies, this provides insight into the potential phospholipid class-specific involvement in AD pathology progression. Overall, our work highlights the relevance and utility of MSI for understanding molecular mechanisms of Alzheimer’s disease.

## Supporting Information Available

AD pathology assessment with H&E and IHC staining; single ion images and ROI statistics of additional sphingo- and phospholipids; and single ion images for all patients and ROI MS spectra for fAD and control brain (PDF)

## Abbreviations

AA: arachidonic acid
AD: Alzheimer’s disease
Aβ: amyloid-β
CerP: ceramide-1-phosphate
DHA: docosahexaenoic acid
fAD: familial Alzheimer’s disease
GM: monosialo-ganglioside
HexCer: ceramide monohexoside
MALDI: matrix-assisted laser desorption/ionization
MSI: mass spectrometry imaging
PE: phosphatidylethanolamine
PE-Cer: ceramide phosphoethanolamine conjugate
PI: phosphoinositol
PSEN: presenilin
PUFA: polyunsaturated fatty acid
ST: sulfatide
