# Supplementary material for: Spatial Neurolipidomics at the Single Amyloid-β Plaque Level in Postmortem Human Alzheimer’s Disease Brain
Source: ACS Chem Neurosci. 2024 Feb 1;15(4):877–88. doi: 10.1021/acschemneuro.4c00006 (PMC10885149; doi:10.1021/acschemneuro.4c00006)
Supplement: Supplementary file 1 — cn4c00006_si_001.pdf [file cn4c00006_si_001.pdf]

Supporting Information

Spatial neurolipidomics at the single amyloid-β plaque level in postmortem human Alzheimer's disease brain

Wojciech Michno[1,2,3,4], Andrew Bowman[5], Durga Jha[1], Karolina Minta[1], Junyue Ge[1], Srinivas Koutarapu[1], Henrik Zetterberg[1,6,7,8,9,10], Kaj Blennow[1,6,11,12], Tammaryn Lashley[7,13], Ron M.A. Heeren[5], Jörg Hanrieder[1,6, 7,14*]

1) Department of Psychiatry and Neurochemistry, Sahlgrenska Academy, University of Gothenburg, Mölndal, Sweden
2) Department of Neuroscience, Physiology and Pharmacology; University College London, London, United Kingdom
3) Department of Public Health and Caring Sciences, Uppsala University, Uppsala, Sweden
4) Science for Life Laboratory (SciLife), Uppsala University, Uppsala, Sweden
5) Maastricht MultiModal Molecular Imaging Institute (M4I), Maastricht University, Maastricht, The Netherlands
6) Clinical Neurochemistry Laboratory, Sahlgrenska University Hospital, Mölndal, Sweden
7) Department of Neurodegenerative Disease, Queen Square Institute of Neurology, University College London, Queen Square, London WC1N 3BG, United Kingdom
8) UK Dementia Research Institute at UCL, London, UK
9) Hong Kong Center for Neurodegenerative Diseases, Clear Water Bay, Hong Kong, China
10) Wisconsin Alzheimer's Disease Research Center, University of Wisconsin School of Medicine and Public Health, University of Wisconsin-Madison, Madison, WI, USA
11) Paris Brain Institute, ICM, Pitié-Salpêtrière Hospital, Sorbonne University, Paris, France
12) Neurodegenerative Disorder Research Center, Division of Life Sciences and Medicine, Department of Neurology, Institute on Aging and Brain Disorders, University of Science and Technology of China and First Affiliated Hospital of USTC, Hefei, P.R. China
13) Queen Square Brain Bank for Neurological Disorders, Department of Clinical and Movement Neurosciences, Institute of Neurology, University College London, London, United Kingdom
14) Science for Life Laboratory (SciLife), University of Gothenburg, Gothenburg, Sweden

* Correspondence: Dr Jörg Hanrieder,

E-mail: jh@gu.se

Content: Supplemental Information Figure S1-S9

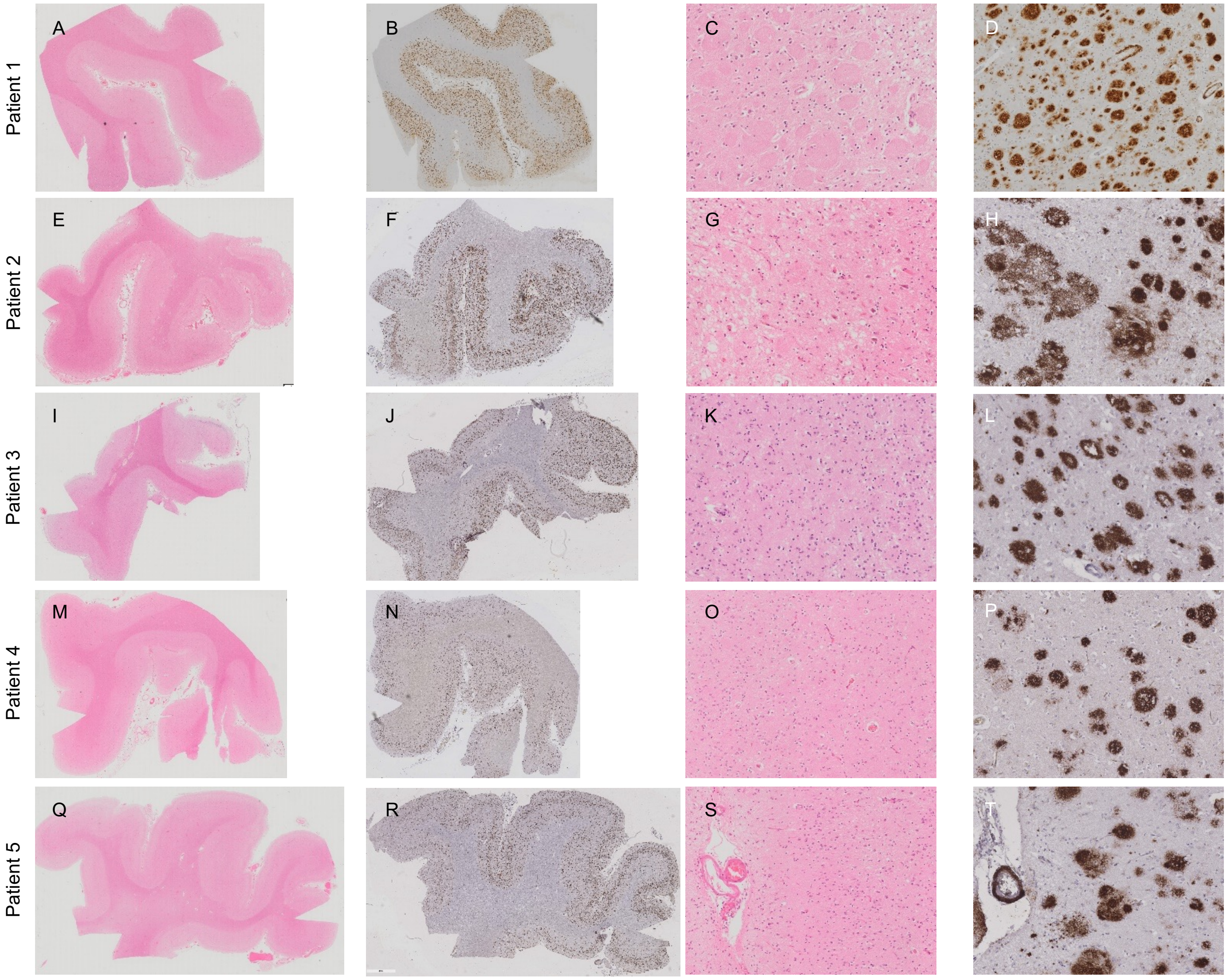

Supplemental Figure 1. Representative overview and magnified images of hematoxylin and eosin (H&E) and Aβ stainings from five patients with *PSEN1* mutations. (A, E, I, M, Q) Overview H&E staining and (B, F, J, N, R) Aβ stainings. Magnified images of (C, G, K, O, S) H&E staining and (D, H, L, P, T) Aβ stainings from the same images, display the presence of Aβ plaques, which are diverse in both shape and size.

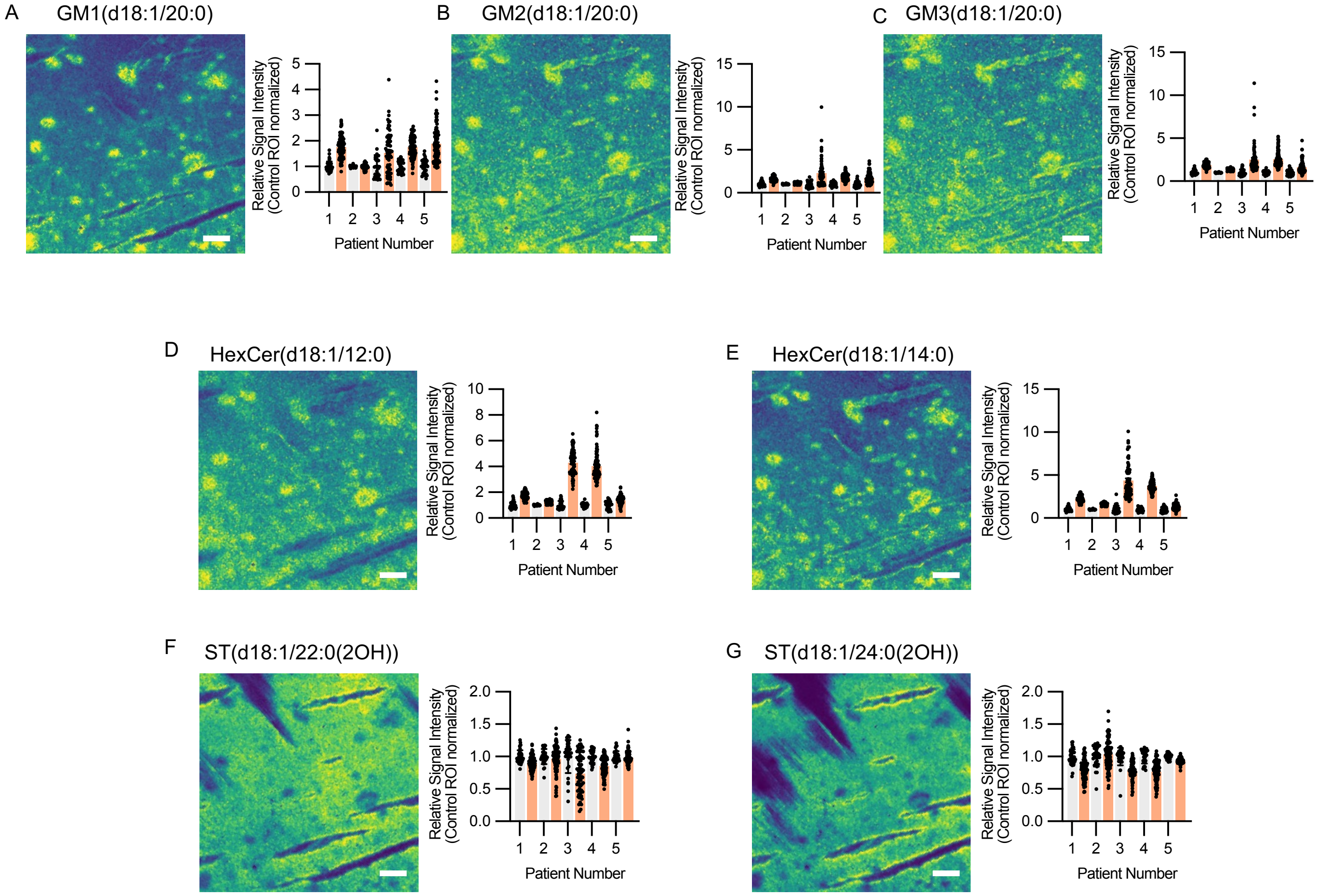

Supplemental Figure 2. MALDI MSI rapifleX data of additional sphingolipids identified in postmortem human AD tissue of five patients with PSEN 1 mutations. Besides the monosialogangliosides (GM) species described in figure 2 we also observed Aβ plaque pathology enrichment of other GM as apparent by the single ion images (left) and relative signal enrichment (right). This included (A) GM1(d18:1/20:0), (B) GM2(d18:1/20:0), and (C) GM3(d18:1/20:0). Additionally, we also observed Aβ plaque pathology specific enrichment of ceramide monohexosides, including (D) HexCer(30:1) and (E) HexCer(32:1). Lastly, the characteristic Aβ plaque associated depletion of sulfatides (ST) seen in Figure 2 is also present for the hydroxylated version of the reported ST species, including including (J) ST(d18:1/22:0) and (K) ST(d18:1/24:0). Signal intensities from ca 100 Aβ plaques and surrounding area was extracted for each patient. Red bar indicates signal from individual Aβ plaques ROIs, gray corresponds to individual control ROIs. Scalebar: 150 µm.

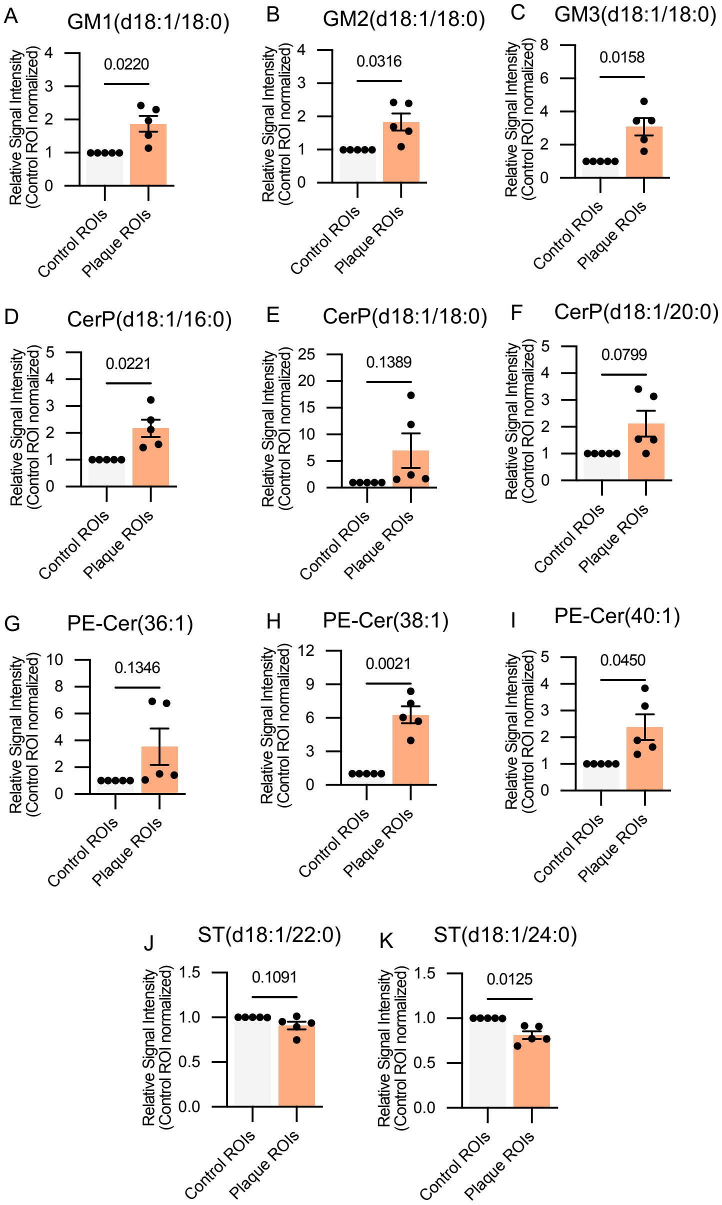

Supplemental Figure 3. Patient average of control ROI and Aβ plaque enriched signal for AD tissue from five patients with *PSEN1* mutations. The Aβ plaque pathology is associated with local enrichment of monosialo-gangliosides (GM), including (A) GM1(d18:1/18:0), (B) GM2(d18:1/18:0), and (C) GM3(d18:1/18:0). Similar Aβ plaque pathology specific enrichment is also present for ceramide-1-phosphates (CerP) and ceramide phosphoethanolamine conjugates (PE-Cer), including (D) CerP (d18:1/16:0), (E) CerP (d18:1/18:0), (F) CerP (d18:1/20:0), respective (G) PE-Cer(36:1), (H) PE-Cer(38:1), (I) PE-Cer(40:1). On the other hand there appears to be a Aβ plaque depletion of sulfatides including (J) ST(d18:1/22:0) and (K) ST(d18:1/24:0). Paired t-test for approximately 100 Aβ plaque or control areas for each of the patient.

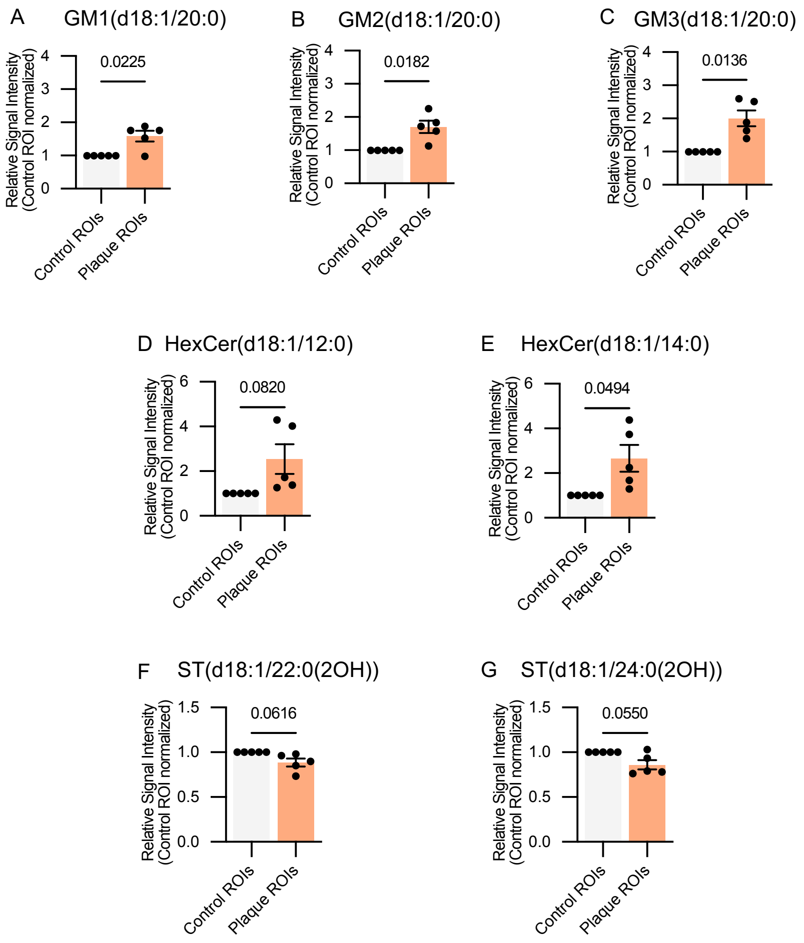

Supplemental Figure 4. Patient average of control ROI and Aβ plaque enriched signal for AD tissue from five patients with *PSEN1* mutations. The Aβ plaque pathology is associated with local enrichment of Besides the monosialo-gangliosides (GM) species described in figure 2 we also observed (A) GM1(d18:1/20:0), (B) GM2(d18:1/20:0), and (C) GM3(d18:1/20:0). Additionally, we also observed Aβ plaque pathology specific enrichment of ceramide monohexosides, including (D) HexCer(30:1) and (E) HexCer(32:1). Lastly, the characteristic Aβ plaque associated depletion of sulfatides (ST) seen in Figure 2 is also present for the hydroxylated version of the reported ST species, including including (J) ST(d18:1/22:0) and (K) ST(d18:1/24:0). Paired t-test for approximately 100 Aβ plaque or control areas for each of the patient.

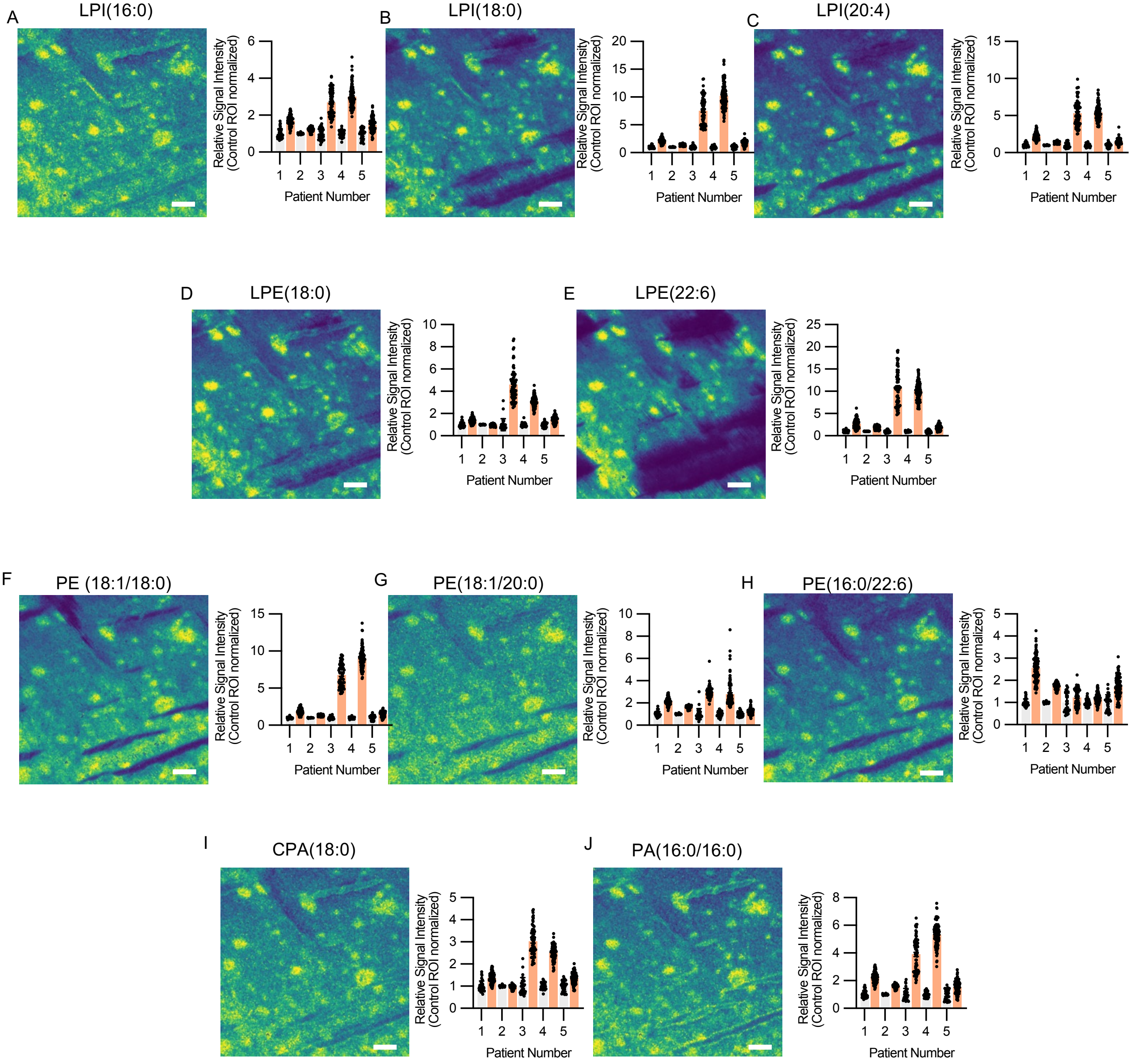

Supplemental Figure 5. MALDI MSI rapifleX data of additional phospholipids identified in postmortem human AD tissue of five patients with PSEN 1 mutations. Besides the arachidonic acid (AA) or docosahexaenoic acid (DHA) containing phospholipids reported in Figure 3, as apparent by the single ion images (left) and relative signal enrichment (right)we identified lyso-phosphatidylinositoles (LPI) such as (A) LPI(16:0), (B) LPI(18:0) and (C) LPI(20:4) - AA. Similar we also observed Aβ plaque pathology specific enrichmentis of lyso-phosphatidylethanolamine (LPE), including (D) LPI (18:0) and (E) LPI (22:6) - DHA, as well as other phosphatidylethanolamine species including (F) PE(18:1/18:0), (G) PE(18:1/20:0), as well as the DHA containing (H) PE(16:0/22:6). Lastly, we also observed cyclic phosphatidic acid (CPA), specifically (I) CPA(18:0), and phosphatidic acid (PA), (J) PA(16:0/16:0). Signal intensities from ca 100 Aβ plaques and surrounding area was extracted for each patient. Red bar indicates signal from individual Aβ plaques ROIs, gray corresponds to individual control ROIs. Scalebar: 150 µm.

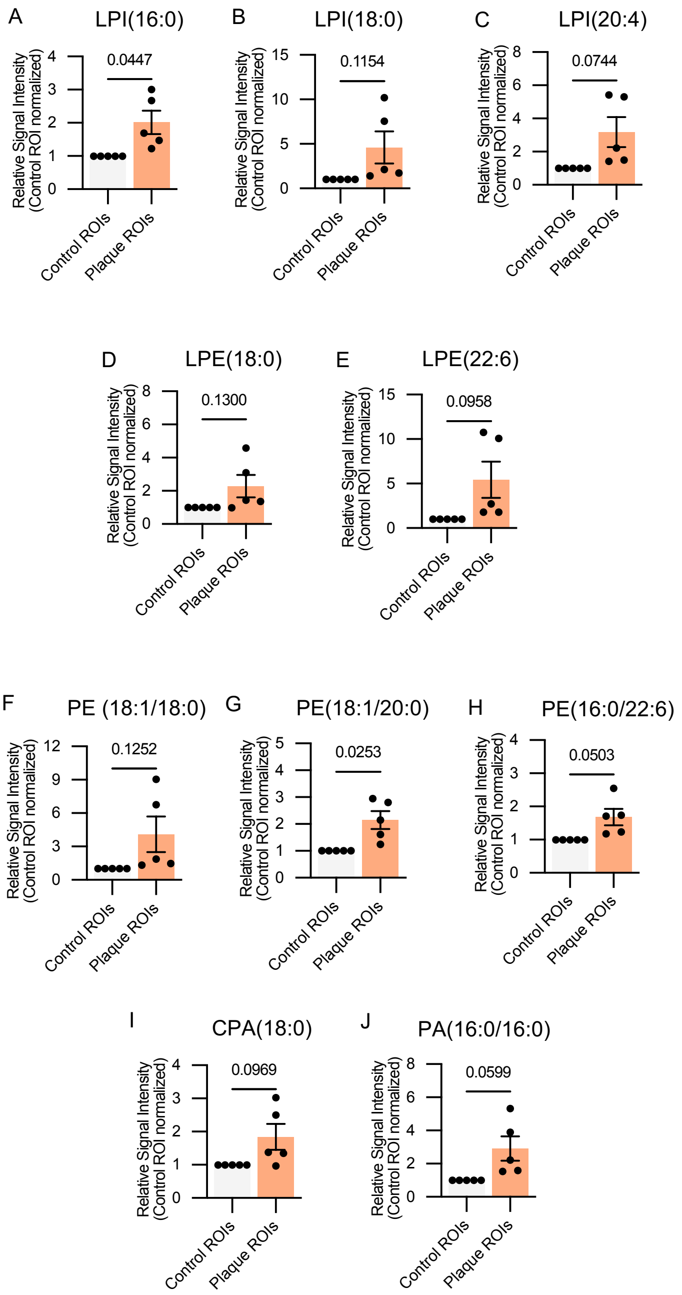

Supplemental Figure 6. Patient average of control ROI and Aβ plaque enriched signal for AD tissue from five patients with *PSEN1* mutations. Besides the arachidonic acid (AA) or docosahexaenoic acid (DHA) containing phospholipids reported in Supplemental Figure 5, as apparent by the single ion images (left) and relative signal enrichment (right)we identified lyso-phosphatidylinositoles (LPI) such as (A) LPI(16:0), (B) LPI(18:0) and (C) LPI(20:4) - AA. Similar we also observed Aβ plaque pathology specific enrichmentis of lyso-phosphatidylethanolamine (LPE), including (D) LPI (18:0) and (E) LPI (22:6) - DHA, as well as other phosphatidylethanolamine species including (F) PE(18:1/18:0), (G) PE(18:1/20:0), as well as the DHA containing (H) PE(16:0/22:6). Lastly, we also observed cyclic phosphatidic acid (CPA), specifically (I) CPA(18:0), and phosphatidic acid (PA), (J) PA(16:0/16:0). Paired t-test for approximately 100 Aβ plaque or control areas for each of the patient.

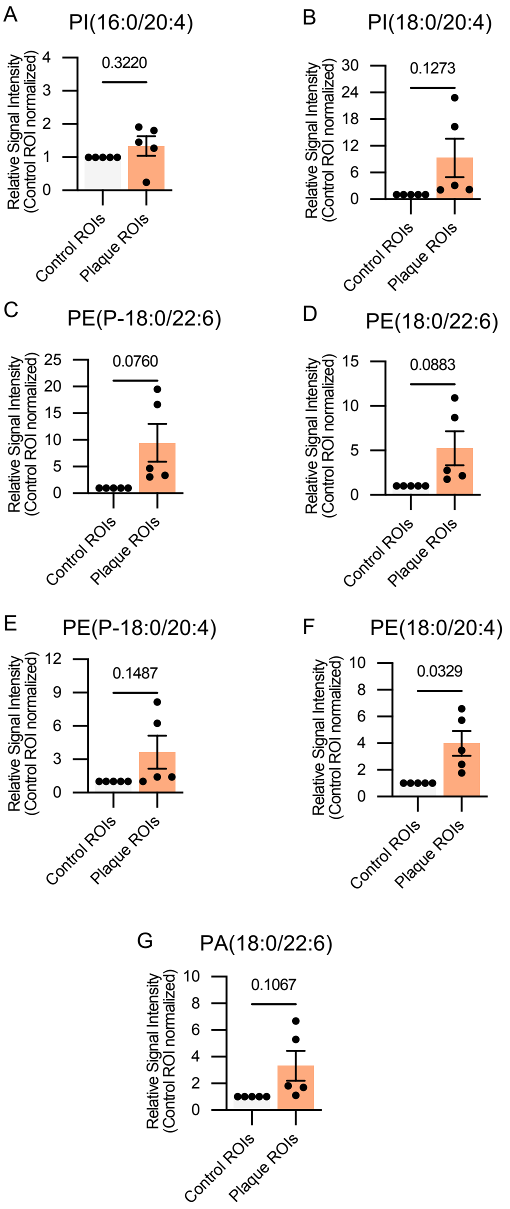

Supplemental Figure 7. Patient average of control ROI and Aβ plaque enriched signal for AD tissue from five patients with *PSEN1* mutations. The Aβ plaque pathology is associated with alterations of arachidonic acid (AA) or docosahexaenoic acid (DHA) containing phospholipids as apparent by the single ion images (left) and corresponding single Aβ plaque relative signal enrichment (right). This includes arachidonic acid (AA) residue containing phosphatidylinositoles, such as (A) PI(16:0/20:4) and (B) PI(18:0/20:4). Similar Aβ plaque pathology specific enrichment is also present for DHA containing phosphatidylethanolamine (C) PE(18:0/22:6) and plasmogen (D) PE(P-18:0/22:6), as well as the AA containing (E) PE(18:0/20:4) and (F) PE(P-18:0/20:4). We also observed enrichment of DHA containing phosphatidic acid, PA(18:0/22:6). Paired t-test for approximately 100 Aβ plaque or control areas for each of the patient.

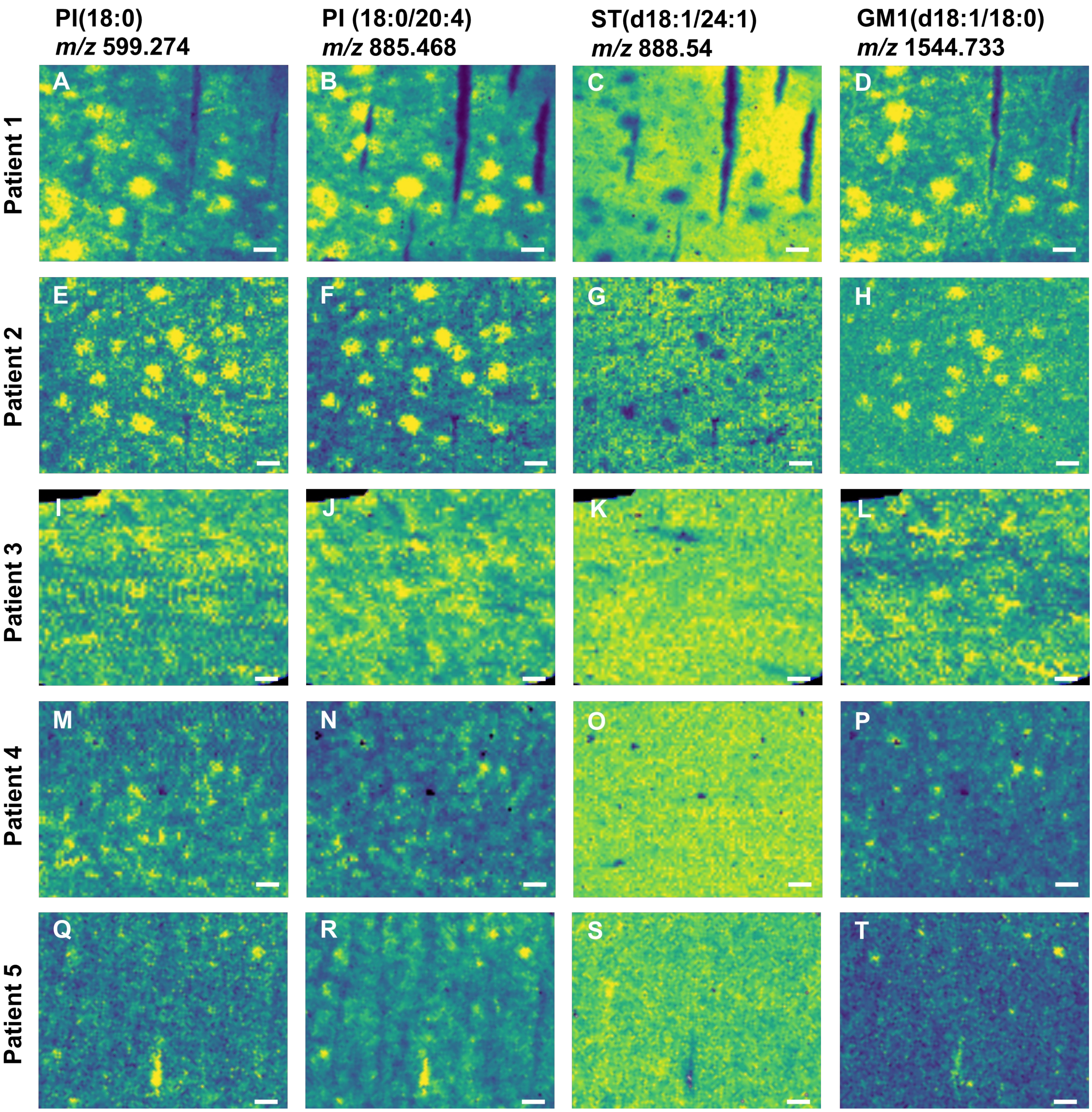

Supplemental Figure 8. Representative MALDI MSI rapifleX data from five PSEN patients. This included (A, E, I, M, Q) PI (18:0), (B, F, J, N, R) PI (18:0/20:4), (C, G, K, O, S) ST (d18:1/24:1), and (D, H, L, P, T) GM1 (d18:1/18:0). Scale bar: 100 µm.

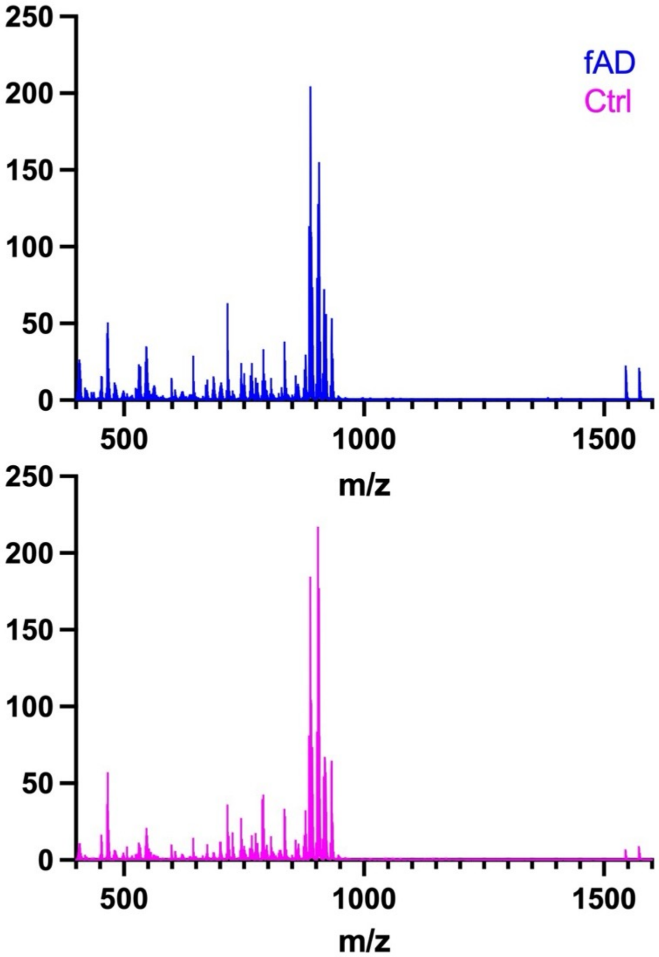

Supplemental Figure 9. Representative sum spectra of MALDI MSI data generated for fAD patients and non-demented control brain.
